# Processing of self-initiated speech-sounds is different in musicians

**DOI:** 10.3389/fnhum.2013.00041

**Published:** 2013-02-22

**Authors:** Cyrill G. M. Ott, Lutz Jäncke

**Affiliations:** ^1^Department of Psychology, Division Neuropsychology, Psychological Institute, University of ZurichZürich, Switzerland; ^2^International Normal Aging and Plasticity Imaging Center, University of ZurichZürich, Switzerland

**Keywords:** motor-induced suppression, musicians, speech, plasticity, internal forward model

## Abstract

Musicians and musically untrained individuals have been shown to differ in a variety of functional brain processes such as auditory analysis and sensorimotor interaction. At the same time, internally operating forward models are assumed to enable the organism to discriminate the sensory outcomes of self-initiated actions from other sensory events by deriving predictions from efference copies of motor commands about forthcoming sensory consequences. As a consequence, sensory responses to stimuli that are triggered by a self-initiated motor act are suppressed relative to the same but externally initiated stimuli, a phenomenon referred to as motor-induced suppression (MIS) of sensory cortical feedback. Moreover, MIS in the auditory domain has been shown to be modulated by the predictability of certain properties such as frequency or stimulus onset. The present study compares auditory processing of predictable and unpredictable self-initiated 0-delay speech sounds and piano tones between musicians and musical laymen by means of an event-related potential (ERP) and topographic pattern analysis (TPA) [microstate analysis or evoked potential (EP) mapping] approach. As in previous research on the topic of MIS, the amplitudes of the auditory event-related potential (AEP) N1 component were significantly attenuated for predictable and unpredictable speech sounds in both experimental groups to a comparable extent. On the other hand, AEP N1 amplitudes were enhanced for unpredictable self-initiated piano tones in both experimental groups similarly and MIS did not develop for predictable self-initiated piano tones at all. The more refined EP mapping revealed that the microstate exhibiting a typical auditory N1-like topography was significantly shorter in musicians when speech sounds and piano tones were self-initiated and predictable. In contrast, non-musicians only exhibited shorter auditory N1-like microstate durations in response to self-initiated and predictable piano tones. Taken together, our findings suggest that besides the known effect of MIS, internally operating forward models also facilitate early acoustic analysis of complex tones by means of faster processing time as indicated by shorter auditory N1-like microstate durations in the first ~200 ms after stimulus onset. In addition, musicians seem to profit from this facilitation also during the analysis of speech sounds as indicated by comparable auditory N1-like microstate duration patterns between speech and piano conditions. In contrast, non-musicians did not show such an effect.

## Background

In the past 15 years, highly trained musicians have become an important model in neuroscience to study the effects of learning-induced cortical plasticity. It is now widely known and accepted that the intensive practice and training needed to achieve high musical skills leads to structural and functional short- and long-term alterations in the brain [for a review see Schlaug (2001); Münte et al. (2002); Jäncke (2009); Shahin (2011)]. There is also growing evidence that these alterations are not restricted particularly to structures and functions associated with perception and production of music, but also affect other domains such as language and speech (e.g., Patel, 2003; Thompson et al., 2003; Schön et al., 2004; Moreno and Besson, 2006; Besson et al., 2007; Marques et al., 2007; Parbery-Clark et al., 2009; Gordon et al., 2010; Schön et al., 2010; Besson et al., 2011; Colombo et al., 2011; Marie et al., 2011; Ott et al., 2011; Patel, 2011; Strait and Kraus, 2011; Kühnis et al., 2012).

However, in order to accomplish a high level of musical skill, the interplay between motor-actions and their resulting sensory consequences plays a critical role and thus comprises a substantial part of the daily training routines performed by adept musicians. On the other hand, various studies have shown that sensory responses to stimuli that are triggered by a self-initiated motor act are suppressed compared with the response to the same stimuli when they are externally triggered (e.g., Weiskrantz et al., 1971; Wolpert and Ghahramani, 2000; Martikainen et al., 2005; Bäss et al., 2008; Aliu et al., 2009; Baess et al., 2009, 2011; Chen et al., 2012). To date, these functional differences are discussed to reflect the neuronal mechanisms providing the ability to discriminate sensory changes caused by one's own actions to those of others, thus enabling successful interaction with a highly complex sensory environment and efficient goal-directed behavior. Having this in mind, the question arises whether the high amount of sensorimotor practice and training that musicians are exposed to also leads to observable alterations with respect to the neural underpinnings of self- vs. externally generated sound discrimination.

Internal forward models of motor control (Wolpert and Kawato, 1998; Wolpert et al., 1998; Wolpert and Ghahramani, 2000; Wolpert and Flanagan, 2001) are addressing this differential processing and have integrated classical biological concepts such as the re-afference principle (Von Holst and Mittelstaedt, 1950; von Holst, 1954) or *corollary discharge* (Sperry, 1950). These models basically assume a prediction mechanism based on motor-to-sensory transformations within the central nervous system, in which an *efference copy* of the motor commands is translated into a representation of the expected sensory event (*corollary discharge*). This prediction encodes some of the expected sensory consequences of the movement to be executed and is then compared through sensory feedback loops to the sensory re-afference. The *efference copy* therefore enables the forward model to accurately predict the expected sensory feedback, resulting in a small prediction error, which in turn translates to a minimal response in the respective sensory cortex. In the absence of such an *efference copy* from the motor system, the forward model is unable to generate an accurate prediction of the sensory feedback, resulting in a larger prediction error and sensory field response, respectively. Moreover, the internal forward model mechanism is assumed to be a dynamic and adaptive modeling process based on estimations of the current state derived from the previous state (Kalveram, 1998, 2000). In other words, the sensory consequences based on the respective motor commands have to be learned by the forward model in order to generate an as accurate as possible prediction.

In human auditory cortex, this sensorimotor interplay can be readily observed. For example, the auditory cortex of a speaker responds to the sound of his own speech with a suppressed activation compared to the activation elicited by passive listening to mere playback of the speech, a phenomenon that has been labeled as *speaking-induced suppression* or SIS (Houde et al., 2002; Eliades and Wang, 2003). It also has been shown that a similar suppression phenomenon occurs in the somatosensory system, where responses to self-produced tactile stimuli are weaker relative to externally generated ones (Blakemore et al., 1998, 2000; Blakemore and Decety, 2001). These similarities suggest that the observed suppression effects reflect a more general property of sensory cortices in the sense that sensory feedback from any motor act is processed by comparing incoming feedback against a respective feedback-prediction derived from an *efference copy* of the motor command that produced the actual sensory feedback. This comparison in turn results in *motor-induced suppression* or MIS (Aliu et al., 2009).

With respect to suppression of auditory cortex activity, there is growing evidence from studies examining arbitrary pairings of a motor act with auditory stimuli. In 1973, Schafer and Marcus showed the human electroencephalographic (EEG) N1-response (peaking at ~100 ms) obtained at the vertex electrode to be significantly smaller to self-triggered auditory stimuli than to identical ones triggered by a machine. More recently, Martikainen et al. (2005) were able to demonstrate by using magnetoencephalography (MEG) that the human supratemporal cortex exhibits significant suppression when responding to self-initiated 1000 Hz sinusoid tones compared to passive listening to the same tones. Baess et al. (2011) used EEG and showed the N1-response to be even more attenuated to self- vs. externally triggered sounds when these were mixed within presentation blocks than the suppression observed in a traditional blocked design. Thus, the N1-suppression effect cannot be simply explained by contextual task differences and rather reflects the involvement of an internal prediction mechanism as stated above. Another study done by Bäss et al. (2008) revealed the N1-suppression effect to be attenuated but still present when sound onset and frequency are unpredictable. In particular, their findings show that forward model mechanisms tolerate uncertainties in the predictability of frequency and onset. In other words, forward models are readily operating as long as a subject is able to identify a sound as self-initiated. This in turn accounts mainly for interactions with unknown objects and/or insufficient visual information about the environment.

Anyhow, to our knowledge there is no study to date that addressed the earlier mentioned question whether intense musical training also leads to observable alterations in the neural underpinnings providing the discrimination of self- vs. externally generated sounds. To investigate this possibility, we compared high-density EEG-recordings of 16 professional pianists to those of 16 musically untrained individuals. We employed a paradigm similar to that used by Bäss et al. in their respective study of 2008, thus applying predictable and unpredictable outcomes of auditory consequences of a button press. Though, we introduced a speech and a piano condition and presented either CV-syllables (“Da” vs. “Ta”; speech condition) or piano tones (“C3” vs. “C5”; piano condition) instead of using pure sinusoid tones as employed in the afore-mentioned papers. The reason for using these stimuli was to elucidate whether possible musical training-induced differences in the magnitude of MIS are restricted to the sounds to which pianists are mainly exposed during practice or if those training effects are more widespread and are also affecting the domain of speech. To modulate the predictability of auditory feedback, the respective sounds were either fixed to the right vs. left response button (predictable outcome) or randomly assigned to the buttons with each button press (unpredictable outcome). By introducing an unpredictable condition, we wanted to verify a finding of one of our previous studies, in which we demonstrated that musicians process voiced and unvoiced stimuli similarly (Ott et al., 2011). According to this, predictability of voicing should affect the magnitude of prediction errors in the forward model of musicians to a lesser degree and thus not attenuate MIS of their auditory cortex to the same extent as of musical laymen.

Taking the most prominent findings of the respective literature into account, we first focused our analysis on amplitude modulations of the classical N1 auditory event-related potential (AEP)-component obtained at the vertex electrode (Schafer and Marcus, 1973; Numminen and Curio, 1999; Numminen et al., 1999; Curio et al., 2000; Houde et al., 2002; Heinks-Maldonado et al., 2005, 2006, 2007; Bäss et al., 2008; Aliu et al., 2009; Ventura et al., 2009; Baess et al., 2011). Here we hypothesized to find stronger suppression of the N1 amplitude in musicians in the predictable condition for speech and piano stimuli since their forward model should generate more precise predictions of the auditory consequences of a motor act due to their intense training, thus leading to smaller prediction errors resulting in stronger MIS. Based on the findings of Ott et al. (2011), we expected CV-syllable induced N1-amplitudes in the unpredictable condition to be comparable to the predictable condition in musicians, whereas N1-suppression should be attenuated in musical laymen when voicing of the syllables is unpredictable. On the other hand, unpredictable piano stimuli should induce attenuation of MIS to a similar extent in both groups.

In a second step, we will also analyze the spatial variations of the scalp voltage distribution across groups and conditions. This approach named Topographic Pattern Analysis (TPA) or evoked potential (EP) mapping has several advantages over conventional ERP analysis techniques. Using this technique we search for time segments of stable map topography that represent functional microstates of the brain. These microstates are assumed to reflect distinct information processing steps and provide particular advantages over the classical ERP analysis, such as experimenter and reference independence [for an overview, see e.g., Murray et al. (2008); Michel et al. (2009); Brunet et al. (2011)]. This kind of analysis exploits the high topographic resolution that high-density EEG-recordings provide. Using this approach, we searched for stable map topographies before, during, and after the N1 time window (similar as in our previous paper, Ott et al., 2011). Here, we are interested in examining whether the duration of these maps are different between musicians and non-musicians in the context of the suppression paradigm.

## Materials and methods

### Subjects

Thirty-two healthy volunteers with normal audiological status and no history of neurological pathology were recruited for this study. Since the focus of this study was not on speech processing *per se* and for the sake of feasibility, we also accepted participants that were indeed german speaking but not native German or Swiss German speakers. However, we did not accept subjects of any tonal mother tongue, such as mandarin or other asian dialects. Sixteen pianists comprised the musician group (12 women, 4 men; mean age ± SD of 25.37 ± 4.44 years), with formal training starting at a mean age ± SD of 6.08 ± 0.94 years. All pianists were students, music teachers and/or members of an orchestra or band and practice piano playing on a daily basis of 1–7 h. The second group consisted of 16 musical laymen (13 women, 3 men; mean age ± SD of 25.12 ± 3.77 years) with no history of piano playing and no formal musical training exceeding the educational context of public elementary and secondary school. Regarding handedness, 4 participants within the pianist group were consistent left-handed, and 12 were consistent right-handed according to the Annett-Handedness-Questionnaire (Annett, 1970). The non-musician group comprised 2 consistent left-handed and 14 consistent right-handed subjects. To determine each participant's degree of musical competence, we applied the “Advanced Measures of Music Audiation” by Gordon (1989) prior to the EEG-experiment. Moreover, all participants also performed a short intelligence test (KAI) in order to rule out significant group differences in intelligence. Table 1 summarizes the descriptive statistics of all criterion measures completed by the participants. All of them were paid for participation and gave informed written consent in accordance with procedures approved by the local ethics committee.

**Table 1:** **Descriptive statistics for the criterion measures completed by the subjects are listed group-wise (M, musicians; NM, non-musicians; GMA, Gordon Musical Aptitude; TS, total score; T, tonal score; R, rhythm score)**.

	**Age**		**Gender**		**IQ**		**GMA**		**Handedness**		
	**Mean**	**SD**	**M**	**F**	**Mean**	**SD**	**Mean**	**SD**	**Left**	**Right**	**Ambi**
M	25.37	4.44	4	12	124.6	6.5	TS^*^: 79.68	12.18	4	12	0
							T^*^: 80.75	14.75			
							R^*^: 77.37	12.65			
NM	25.12	3.77	3	13	128.4	11.9	TS^*^: 54.87	20.95	2	14	0
							T^*^: 55.25	20.72			
							R^*^: 55.37	22.00			

* *Difference between experimental groups is significant at p* < *0.001 [T*_*TS*_*(31)* = *4.095*; *T*_*T*_*(31)* = *4.009*; *T*_*R*_*(31)* = *3.467*; *note that T-Tests were only applied to variables “Age,” “IQ,” and “GMA.” For “Gender” and “Handedness”, Chi-Square tests were used]. All statistical tests shown in this table were conducted comparing the two experimental groups*.

### Stimuli

In this experiment, all participants either heard consonant-vowel (CV) syllables or piano tones. The CV-syllables consisted of a subset of speech stimuli already used in previous studies, such as Jäncke et al. (2002), Meyer et al. (2007), and Ott et al. (2011). These syllables (/da/, /ta/) were digitally recorded by a trained phonetician at a sampling depth of 16-bit and a sampling rate of 44.1 kHz. Onset, duration, intensity, and fundamental frequency of the syllables were synchronized and edited by means of a speech editor. The articulatory release formed the criterion for temporal alignment of the syllables. Depending on their voice-onset-time (VOT), the duration of the stimuli ranged from 310 to 360 ms with a vowel duration of 300 ms (VOT's in ms for the stops were approximately “d” = 04 and “t” = 49). The piano stimuli comprised two piano tones of different pitch (/C3/, fundamental frequency at 130,813 Hz and /C5/, fundamental frequency at 523,251 Hz) generated with the Logic Express® software (Version 9.1.7, Apple® Inc; 95014 Cupertino, CA; http://www.apple.com/logicpro/). The duration of the tones was set to 360 ms. All auditory stimuli were presented binaurally at a sound pressure level of about 75 dB SPL using hi-fi headphones. Stimulus application and response recording was done using Presentation® software (Neurobehavioral Systems, USA).

### Experimental setup

Based on previous research on self-initiated sounds (Schafer and Marcus, 1973; Martikainen et al., 2005; Bäss et al., 2008; Baess et al., 2011), we introduced three different tasks and a preceding training trial in our experiment:

In the motor-auditory (MA) task, subjects were instructed to generate an incidental key-press sequence by randomly pressing one or another out of two buttons on a computer keyboard with their left and right index fingers. Speech and piano stimuli were presented separately in three blocks at a time. Thus, each button press immediately triggered either a CV-syllable or a piano tone (0-delay stimulus application), respectively. The voiced CV-syllable (/da/) and the low-pitch piano tone (/C3/) were assigned to the left, whereas the unvoiced CV-syllable (/ta/) and the high-pitch piano tone (/C5/) were assigned to the right button. This assignment was not counterbalanced across subjects, as hearing a high piano tone after a left index finger and a low tone after a right index finger button press would be perceived as fairly disturbing, especially by pianists. To maintain consistency between speech and piano stimuli, these assignments were retained accordingly for both stimulus types. The subjects were asked to listen attentively to the sounds that were triggered by them. The interval between button presses was self-paced at a rate of about once every 3.5 s. Subjects also determined the sequential order of left vs. right button presses by themselves and were told to avoid estimating the intervals by internally counting seconds. Each button press was recorded to produce a trigger sequence for subsequent use in the auditory-only (A) task. Moreover, the Presentation® software counted the button presses in real-time and automatically ceased an ongoing block as soon as a minimum of 30 button presses per side (left vs. right index fingers) was reached. This resulted in a total of at least 90 self-initiated auditory stimulations with each CV-syllable and piano tone.

In the A task, the subjects listened to “externally” generated sequences of CV-syllables or piano tones, which used their own respective trigger sequences from the MA task. Using this approach, stimulation in the A task was identical to the MA task.

In the motor-only (M) task, subjects were again instructed to press the respective buttons in a self-paced interval and self-determined sequential order as in the MA task, but no sounds were delivered subsequently. This task served as a control condition to rule out the possibility of mere motor activity being responsible for any observed differences between the tasks involving auditory processing and between experimental groups as well. Hence, the M-task was subtracted from the other two tasks involving motor action prior to any further analysis.

To achieve unpredictability of the sensory consequence of a button press in the MA-unpredictable task (MAU), the respective assignments between buttons and auditory stimuli were randomly shuffled after each button press. Thus, the onset of a certain stimulus remained predictable (0-delay), whereas voicing of the CV-syllables (/da/ vs. /ta/) and pitch of the piano tones (/C3/ vs. /C5/) became unpredictable, respectively. Again, speech and piano stimuli were presented separately in three blocks at a time and the Presentation® software automatically ceased ongoing blocks after 30 self-paced/self-sequenced button presses per side.

Prior to the EEG-recordings, participants were trained to perform the self-paced rate of 3.5 s with visual feedback indicating whether their rate was too slow (>4.5 s), too fast (<2.5 s), or just right (2.5–4.5 s). During the experimental blocks, no visual feedback about their performance was given at all. As described, each task (MA, A, M, and MAU) comprised three blocks containing at least 60 button presses (30 left and 30 right) and/or auditory stimulations. All tasks were performed separately with speech and piano stimuli, resulting in a total amount of 1440 trials per subject. All blocks were presented in a randomized sequence over all participants. Though, as stimulation in the A task was dependent on the acoustical stimulation in the MA task, A task blocks could never precede their corresponding MA task blocks.

### AEP recordings

Due to the fairly long duration of the experiment, we decided to split EEG-data acquisition in two separate recording sessions. EEG was recorded using a high-density Geodesic EEG system® (GSN300; Electrical Geodesic Inc., Oregon; http://www.egi.com) with 256-Channel HydroCel Geodesic Sensor Nets® (HCGSN120). Data was sampled at 500 Hz and band-pass filtered at 0.1–100 Hz. The vertex electrode (Cz) served as on-line recording reference and its exact location on each subject's scalp was noted down in order to ensure a preferably identical placement of the sensor net in the two recording sessions. Impedance was kept below 30 kOhm. During EEG recording, participants sat in a shielded, dimly lit room and a fixation cross was presented on an LCD-screen in front of them in order to reduce eye movements.

### Data analysis—EEG, N1 component analysis

Each participant's EEG recordings were imported and analyzed in the BrainVision Analyzer2 software (Version 2.0.1; Brain Products GmbH, D-82205 Gilching; http://www.brainproducts.com). In a first step, data was band-pass filtered at 1–30 Hz. An ICA (independent component analysis) was then performed to correct for ocular artifacts (e.g., Jung et al., 2000). Each EEG recording was visually inspected and trials with sweating and muscle artifacts, amplifier saturation, and remaining eye blinks or eye movements were excluded by means of a fully automatic raw data inspector. Noisy channels were interpolated and the data was then re-referenced to linked mastoid electrodes for ERP calculation. Each ERP waveform was an average of more than 60 repetitions of the EEG sweeps within a certain task evoked by the same auditory stimulus (MA, A, and MAU tasks) or button press (M task), respectively. EEG recordings were sectioned into 600 ms segments (200 ms pre-stimulus and 400 ms post-stimulus) and a baseline correction using the signal's pre-stimulus portion was carried out. To correct for mere motor activity in the MA and MAU tasks, difference waves between MA/MAU and M tasks were computed subsequently. Finally, ERP's for each stimulus and task were averaged for each subject and grand-averaged across subjects within the two groups separately.

In order to statistically confirm relevant differences between AEP's at Cz as a function of experimental group, task, and stimulus, mean amplitude event-related potentials (ERP's) time-locked to the auditory stimulation were measured in a specific latency window. This was individually pre-determined for each subject, task, and stimulus by visual inspection of the event-related signal. These individual latency windows were centered at the peak of the prominent N1 component and covered a total signal length of 10 ms around the center. Individually chosen latency windows were used to ensure that the mean amplitudes actually reflect the N1 peak values of every subject, task, and stimulus. Mean amplitudes were then averaged separately within groups (i.e., “pianists” vs. “non-musicians”), depending on the respective task (MA, A, MAU) and stimulus (/da/ vs. /ta/ and /C3/ vs. /C5/). Subsequently, a 2 × 3 × 2 repeated measure Analysis of Variance (ANOVA) with a between-subject factor (*group*) and two within-subjects factors (*task* and *voiceness/pitch*) was computed for the central electrode (Cz). We used the multivariate approach to handle the problem of heteroscedasticity in repeated measurement designs (O'Brien and Kaiser, 1985). Thus, we will report *F*-values estimated from the multivariate Wilks' lambda statistic computed within the MANOVA. Subsequently, Bonferroni-Holm adjusted *post-hoc t*-tests were applied. The global level of significance was set at *p* < 0.05 for all statistical analyses.

### Data analysis—EEG, topographic pattern analysis (TPA)

TPA was performed for speech and piano stimuli separately. All subject- and grand-averaged ERP's were imported into the Cartool software (Version 3.51; The Cartool Community group, https://sites.google.com/site/cartoolcommunity/) and recalculated against the average reference. Then, the dominant map topographies on the scalp were identified by defining segments of stable voltage topography (or EP maps) using a topographic atomize and agglomerate hierarchical cluster analysis (T-AAHC) in the grand-averaged ERP's across tasks, stimuli, and groups over the full EEG segment length of 400 ms. These template maps are the mean maps over the period where the stable voltage topography segments were found. The clustering does not account for the latencies of maps, but only for their topographies and is done as a hypothesis generation step wherein the sequence of template maps that best accounts for the data is identified (Murray et al., 2008). The combination of a modified cross-validation and the Krzanowski–Lai criterion (e.g., Pascual-Marqui et al., 1995; Murray et al., 2008) was then used to determine the optimal number of templates. In accordance with the Cartool user guidelines (The Cartool Community group, https://sites.google.com/site/cartoolcommunity/user-s-guide), we identified the first on- and last offset of the typical N1-like map across tasks and groups by using the landscape obtained by the segmentation process. For speech stimuli, first on- and last offset of this map were at 0 and 192 ms, for piano stimuli they were at 0 and 202 ms. In a next step, we statistically verified the presence of each map that was found in the group-averaged data within that particular epoch over the same period in the ERP's of the individual subjects (i.e., “single-subject fitting”; Murray et al., 2008). This procedure is based on the spatial correlation between the template maps and the single-subject ERP data. This step allowed us to determine the duration of any given template map for each condition within the pianist and non-musician group between 0 and 192 ms (speech) and 0 and 202 ms (piano), respectively. These duration values were then statistically evaluated for each map of interest by means of a 2 × 3 × 2 repeated measure ANOVA with the factors *group* (between-subjects), *task*, and *voicing/pitch* (within-subjects), as in the classical N1 component analysis. Subsequently, any significant interactions were further examined by applying Bonferroni–Holm adjusted *post-hoc t*-tests.

## Results

### EEG data—N1 component analysis (peak amplitudes)—speech

**Figure 1:**
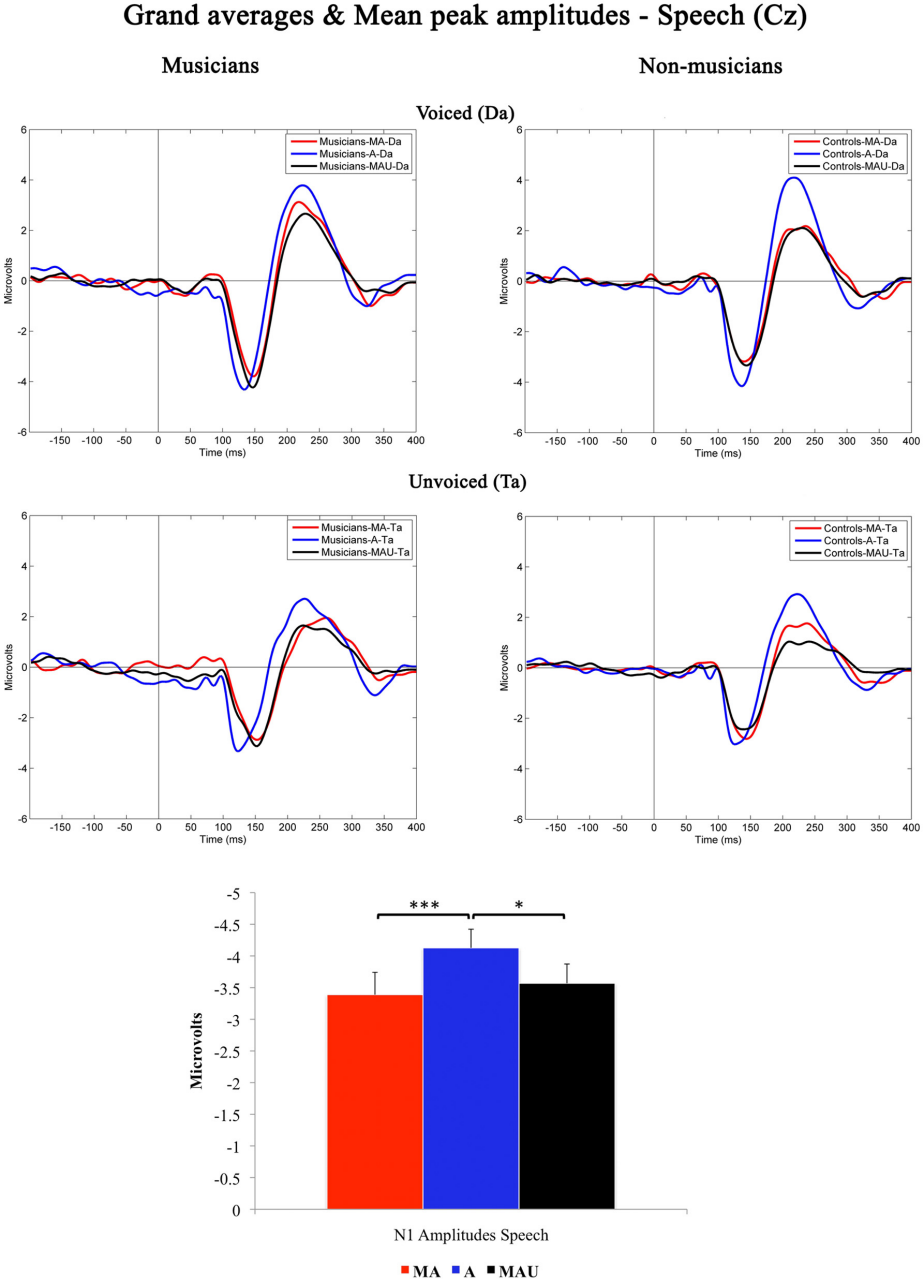
**Grand-averaged waveforms and corresponding mean N1 peak amplitudes evoked by voiced (top) and unvoiced (bottom) CV-syllables recorded at Cz for musicians (left) and non-musicians (right).** MA, Motor-Auditory task; A, Auditory-Only task; MAU, Motor-Auditory unpredictable task. Mean N1 peak amplitudes are displayed irrespective of experimental group. ^*^Difference is significant at *p* < 0.05, *T*_(31)_ = −2.379. ^***^Difference is significant at *p* < 0.001, *T*_(31)_ = −3.433. Error bars indicate standard errors. Time point “0” refers to the stimulus onset.

Grand-averaged waveforms evoked by CV-syllables recorded at Cz and the respective mean N1 peak amplitudes are illustrated in Figure 1. All stimuli elicited a prominent N1 component peaking at ~150 ms. Results of the 2 × 3 × 2 repeated measures ANOVA revealed a significant main effect for the factor *task* [*F*_(1, 30)_ = 6.974, *p* < 0.05].

Bonferroni–Holm adjusted *post-hoc t*-tests indicated smaller amplitudes in the MA [*T*_(31)_ = −3.433, *p* < 0.001] and MAU [*T*_(31)_ = −2.379, *p* < 0.05] tasks compared to the A task. The difference between the MA and MAU tasks failed to reach significance [*T*_(31)_ = 1.035, *p* > 0.05]. Thus, a distinct attenuation to self-relative to externally initiated speech sounds was observed in general, replicating the results of various other studies addressing MIS and SIS, respectively (e.g., Schafer and Marcus, 1973; Houde et al., 2002; Eliades and Wang, 2003; Martikainen et al., 2005; Bäss et al., 2008; Baess et al., 2011).

Moreover, a significant main effect for the factor *voicing* was found in the ANOVA [*F*_(1, 30)_ = 40.362, *p* < 0.001], pointing to smaller amplitudes evoked by the unvoiced CV-syllable /ta/ than by the voiced CV-syllable /da/. Again, this finding is in line with the pre-existing literature covering the topic of sub-segmental speech processing (e.g., Simos et al., 1998; Sharma et al., 2000; Zaehle et al., 2007; Ott et al., 2011). However, neither significant interactions nor group-related effects were found with respect to N1 peak amplitudes elicited by speech.

### EEG data—N1 component analysis (peak amplitudes)—piano

**Figure 2:**
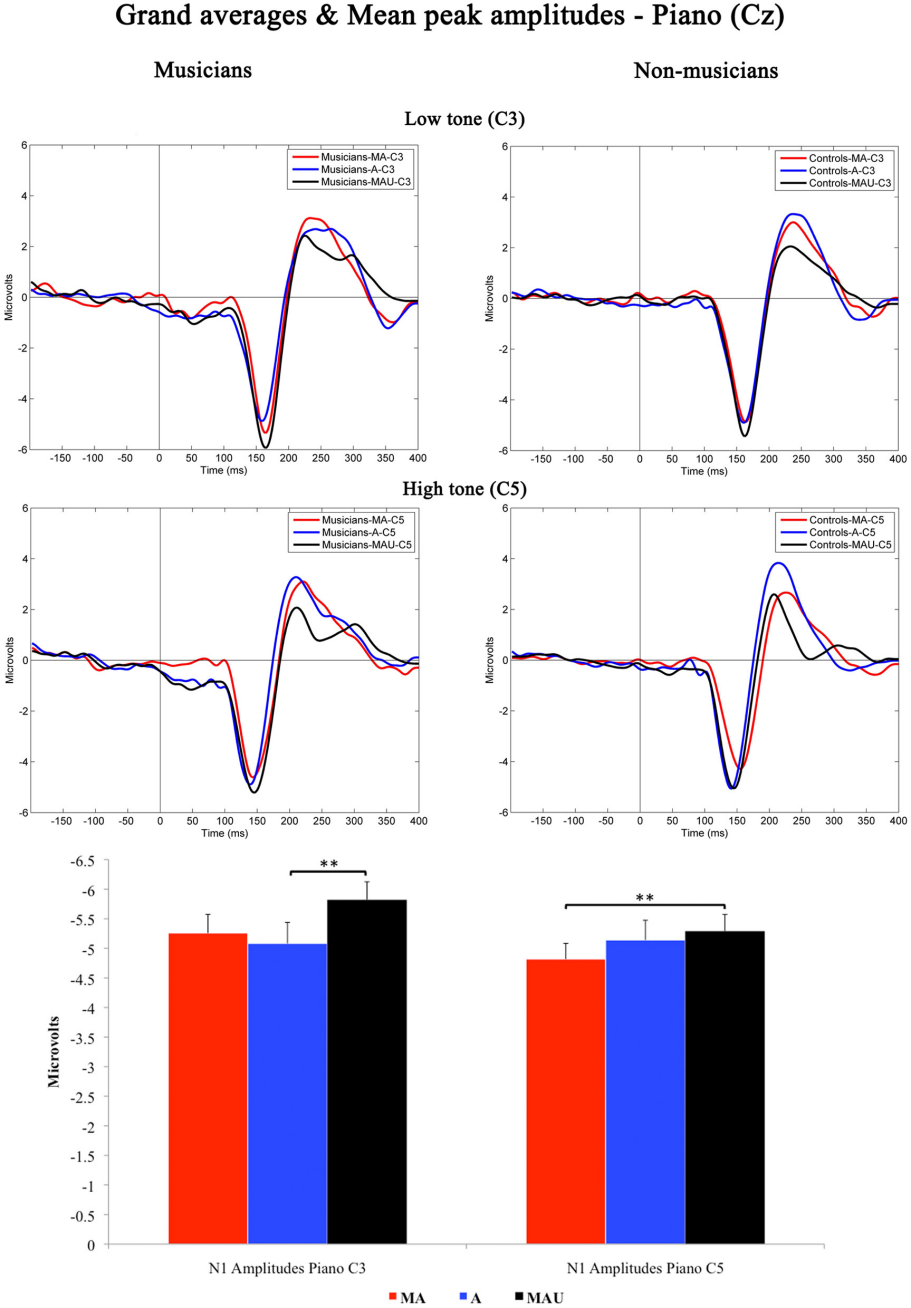
**Grand-averaged waveforms and corresponding mean N1 peak amplitudes evoked by low (top) and high (bottom) piano tones recorded at Cz for musicians (left) and non-musicians (right).** MA, Motor-Auditory task; A, Auditory-Only task; MAU, Motor-Auditory unpredictable task. Mean N1 peak are displayed for low (left) and high (right) piano tones separately and irrespective of experimental group. ^**^Differences are significant at *p* < 0.01, *T*_(31)_ = 3.055 and *p* < 0.01, *T*_(31)_ = 2.747, respectively. Error bars indicate standard errors. Time point “0” refers to the stimulus onset.

Figure 2 shows the grand-averaged waveforms obtained at Cz elicited by low and high piano stimuli and the according mean N1 peak amplitudes. As found for CV-syllables, all piano stimuli evoked a prominent N1 component peaking at ~150 ms. The 2 × 3 × 2 repeated measures ANOVA yielded significant main effects for the factors *task* [*F*_(1, 30)_ = 4.408, *p* < 0.05] and *pitch* [*F*_(1, 30)_ = 2.129, *p* < 0.05]. In addition, a significant interaction *task* by *pitch* [*F*_(1, 30)_ = 4.426, *p* < 0.05] was revealed. With respect to group-related differences regarding N1 peak amplitudes elicited by piano sounds, no significant effects were found.

Subsequent *post-hoc* analyses were applied for low and high piano tones separately, using Bonferroni–Holm adjusted *t*-tests. Low piano tones-evoked stronger N1 potentials in the MAU task relative to the A task [*T*_(31)_ = 3.055, *p* < 0.01], whereas high piano tones-evoked stronger potentials in the MAU task relative to the MA task [*T*_(31)_ = 2.747, *p* < 0.01]. All other comparisons failed to reach significance. Thus, no significant attenuation of mean N1 peak amplitudes to self-initiated piano sounds was observed.

### EEG data—topographic pattern analysis—speech

**Figure 3:**
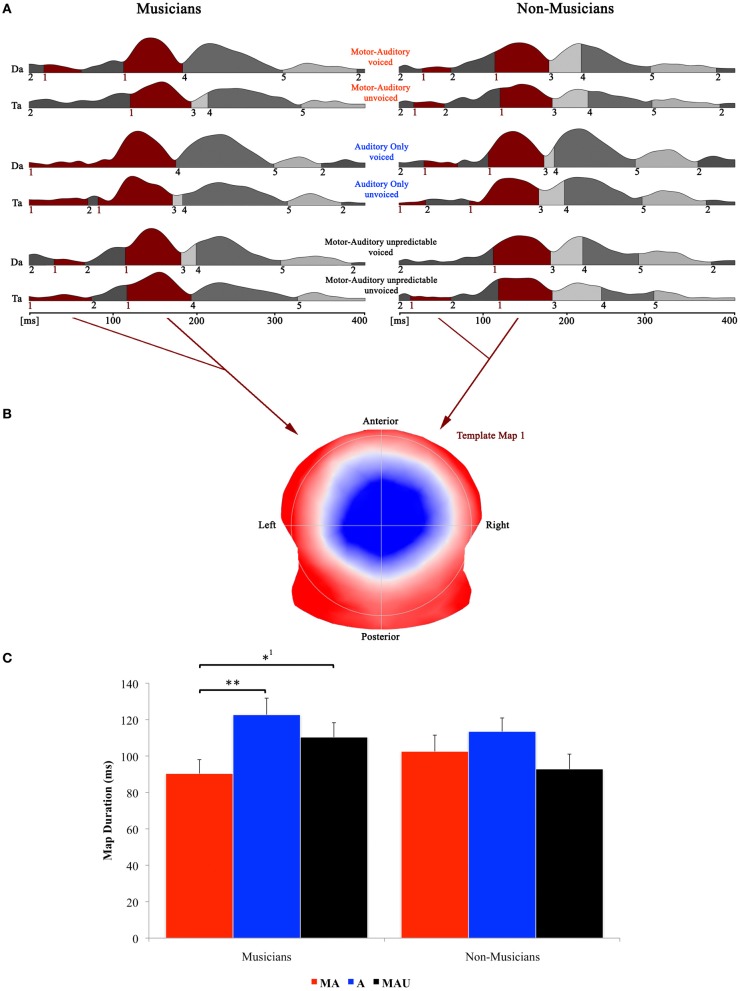
**Evoked potential data; speech condition. (A)** Segments of stable map topography are shown group wise (left: musicians, right: non-musicians) for each condition under the global field power curve from 0 to 400 ms. The numbers below the segmentation landscape indicate the different template maps present in the grand-averaged data. Auditory N1-like template map 1 was found between 0 and 192 ms and was significantly shorter in the motor-auditory (MA) relative to the auditory-only (A) and motor-auditory unpredictable (MAU) tasks within the musician group. In the non-musician group, durations were comparable. **(B)** Map topography. Blue regions indicate negative, red regions indicate positive potential fields. **(C)** Duration of auditory N1-like template map (map 1) for musicians (left) and non-musicians (right) in the respective tasks. Durations are indicated irrespective of voicing. ^**^Difference is significant at *p* < 0.01, *T*_(15)_ = 3.322. ^*1^Difference is significant at *p* < 0.05, *T*_(15)_ = −2.214. Note that the difference indicated with ^*1^ failed to reach significance after correction for multiple comparisons. Also note that the data shown in panels (**A** and **B**) are derived of grand averaged waveforms, whereas map durations shown in panel **(C)** are obtained from individual ERPs. Scaling of template maps is normalized to values between −1 and 1 (for a detailed description of the microstate analysis procedure see e.g., Murray et al., 2008).

Figure 3 shows the speech-related results of the topographical EP mapping of the grand-averaged data for each group and task. Following the Cartool user guidelines (The Cartool Community group, https://sites.google.com/site/cartoolcommunity/user-s-guide), we first visually inspected the landscape and template maps resulting from the segmentation process to determine the particular time period and maps for the single-subject fitting. In order to provide consistency with the examination of the classical N1 component, we focused our analyses to the one template map that expressed a typical N1-like auditory topography (map 1) and therefore chose the time period for the single-subject fitting according to the first on- and last offset of this particular map. This resulted in a time window for the fitting procedure of 0–192 ms. Duration values of this map were then extracted from the fitted single-subject data in terms of timeframes and compared by means of a repeated measure ANOVA.

Results of the 2 × 3 × 2 repeated measure ANOVA with between-subjects factor *group* and within-subjects factors *task* and *voicing* yielded a significant main effect for the factor *task* [*F*_(1, 30)_ = 3.928, *p* < 0.05] and a significant *task* by *group* interaction [*F*_(1, 30)_ = 3.424, *p* < 0.05].

Separate *post-hoc* analyses were conducted for the two experimental groups, using Bonferroni–Holm adjusted *t*-tests. Since no significant effect for the factor *voicing* was found, the respective auditory N1-like map (map 1) durations for the two CV-syllables (/da/ vs. /ta/) were averaged and analyzed together. *Post-hoc t-tests* revealed significantly longer durations for map 1 in the A task relative to the MA task in the pianist group [*T*_(15)_ = 3.322, *p* < 0.01]. Moreover, map 1 durations were longer in the MAU task compared to the MA task in the same group [*T*_(15)_ = −2.214, *p* < 0.05], though this particular difference failed to reach significance after correction for multiple comparisons. On the other hand, no significant differences between tasks regarding auditory N1-like map durations were found in the non-musician group at all.

### EEG data—topographic pattern analysis—piano

**Figure 4:**
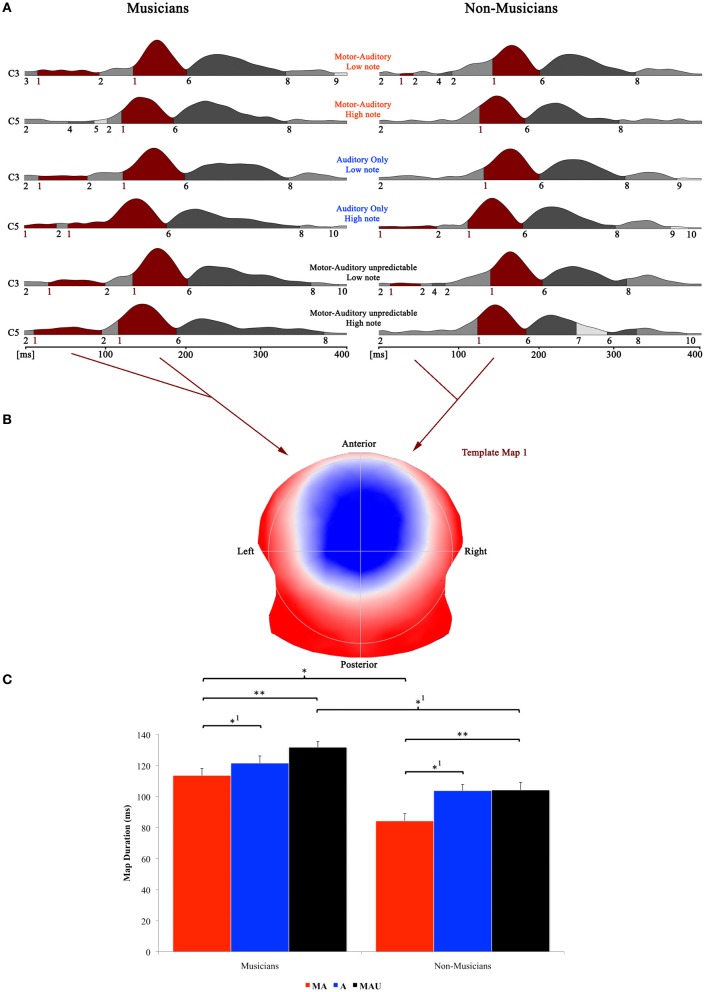
**Evoked potential data; speech condition. (A)** Segments of stable map topography are shown group wise (left: musicians, right: non-musicians) for each condition under the global field power curve from 0 to 400 ms. The numbers below the segmentation landscape indicate the different template maps present in the grand-averaged data. Auditory N1-like template map 1 was found between 0 and 202 ms and was significantly longer in the motor-auditory unpredictable (MAU) relative to the motor-auditory (MA) task in both experimental groups. Map 1 durations were also longer in the auditory-only (A) relative to the MA task in both groups. In addition, musicians exhibited longer map 1 durations in the MA and MAU tasks compared to non-musicians. **(B)** Map topography. Blue regions indicate negative, red regions indicate positive potential fields. **(C)** Duration of auditory N1-like template map (map 1) for musicians (left) and non-musicians (right) in the respective tasks. Durations are indicated irrespective of pitch. ^**^Difference is significant at, *p* < 0.01, *T*_(31)_ = −2.744. ^*1^Difference is significant at *p* < 0.05, *T*_(31)_ = 1.719 (MA vs. A) and *T*_(31)_ = 1.766 (MAU_musicians_ vs. MAU_non-musicians_). ^*^Difference is significant at *p* < 0.05, *T*_(31)_ = 2.099. Note that the difference indicated with ^*1^ failed to reach significance after correction for multiple comparisons. Also note that the data shown in panels (**A** and **B**) are derived of grand averaged waveforms, whereas map durations shown in panel **(C)** are obtained from individual ERPs. Scaling of template maps is normalized to values between −1 and 1 (for a detailed description of the microstate analysis procedure see e.g., Murray et al., 2008).

Piano-tone related results of the topographical EP mapping are illustrated in Figure 4. The same procedure as with speech stimuli was used for the analysis. All analyses were again focused on the template map exhibiting a typical auditory N1-like topography (map 1) and the time window for the single-subject fitting thus ranged from 0 to 202 ms in accordance with first on- and last offsets of this particular map.

Here, the 2 × 3 × 2 repeated measures ANOVA yielded significant main effects for the factors *task* [*F*_(1, 30)_ = 3.832, *p* < 0.05] and *group* [*F*_(1, 30)_ = 4.572, *p* < 0.05]. *Post-hoc t*-tests revealed significantly longer durations for map 1 in the MAU relative to the MA task [*T*_(31)_ = −2.744, *p* < 0.01] and in the A task relative to the MA task [*T*_(31)_ = 1.719, *p* < 0.05]. Though, the latter comparison failed to reach significance after correction for multiple comparisons. In addition, musicians exhibited longer auditory N1-like map durations in the MA and MAU tasks compared to non-musicians [*T*_(31)_ = 2.099, *p* < 0.05 and *T*_(31)_ = 1.766, *p* < 0.05, respectively]. Again, the latter comparison did not reach significance after correcting for multiple comparisons. Other than that, neither further main effects nor interactions were found.

## Discussion

The purpose of this study was to examine the extent to which intense musical training leads to functional alterations with respect to the neural underpinnings of the discrimination between self- vs. externally initiated sounds, a phenomenon known as *MIS* (e.g., Weiskrantz et al., 1971; Schafer and Marcus, 1973; Numminen and Curio, 1999; Numminen et al., 1999; Curio et al., 2000; Wolpert and Ghahramani, 2000; Houde et al., 2002; Heinks-Maldonado et al., 2005; Martikainen et al., 2005; Heinks-Maldonado et al., 2006, 2007; Ford et al., 2007; Bäss et al., 2008; Aliu et al., 2009; Baess et al., 2009, 2011; Chen et al., 2012). We were specifically interested in whether possible musical training-induced alterations were restricted to the sounds to which musicians are mainly exposed during their daily practice routines or if they are more widespread and are also affecting the processing of speech. Our hypothesis was that musical practice would have shaped the forward model of musicians in general such that it generates more precise predictions derived of motor-to-sensory transformations leading to smaller prediction errors and hence enhanced MIS of the auditory cortex. Based on the pre-existing literature addressing the topic of MIS in the auditory domain (e.g., Martikainen et al., 2005; Bäss et al., 2008; Aliu et al., 2009; Baess et al., 2009, 2011), we focused our analyses on the early auditory processing steps within the first 200 ms of stimulus onset and anticipated different neurophysiological activation patterns during these early processing stages.

In this study we used voiced and unvoiced CV-syllables (/da/ vs. /ta/, speech condition) and low- and high-pitched piano tones (/C3/ vs. /C5/, piano condition). Furthermore, we introduced a task in which the particular sensory consequence of a certain motor-action was unpredictable in terms of voicing (speech condition) or pitch (piano condition). To control for mere motor activity possibly being responsible for any observed suppression effects involving auditory processing, a M task was also applied and corresponding difference waves between MA and M tasks were calculated for further analysis. High-density EEG recordings were derived during the early acoustic processing stages and analyzed in two different ways: Firstly we calculated and analyzed conventional AEP components and focused on the N1 component, which is particularly susceptible for MIS-effects (e.g., Schafer and Marcus, 1973; Numminen and Curio, 1999; Numminen et al., 1999; Curio et al., 2000; Houde et al., 2002; Heinks-Maldonado et al., 2005, 2006, 2007; Martikainen et al., 2005; Ford et al., 2007; Bäss et al., 2008; Aliu et al., 2009; Ventura et al., 2009; Baess et al., 2011). Complementary to the conventional ERP analysis we also applied a TPA. With this method we take advantage of the entire spatial information of our high-density EEG recording and recover a more detailed activation pattern. Using this approach, we focused on the microstate exhibiting a typical auditory N1-like topography and corresponding time windows of 0–192 ms (speech condition) and 0–202 ms (piano condition), respectively. As microstates and time windows are identified in an entirely data-driven approach substantially decreasing the subjective influence on the data analysis, this method can be considered a more objective method to analyze evoked electrical responses (Michel et al., 2009; Brunet et al., 2011).

### Speech condition

With respect to CV-syllables, we found that the N1 amplitude is significantly attenuated to self-initiated relative to externally initiated sounds in general, which is in line with the pre-existing literature covering MIS and SIS in particular (e.g., Houde et al., 2002; Eliades and Wang, 2003). Moreover, suppression of the N1 component is slightly reduced in the MAU relative to the MA task such as reported by Bäss et al. (2008), though the direct comparison between the two tasks failed to reach significance. In addition, durations of the microstate exhibiting a typical auditory N1-like topography are significantly shorter during early auditory processing stages (i.e., 0–192 ms) in the pianist group when CV-syllables are self-initiated and their outcome in terms of voicing is predictable compared to when they are externally initiated or voicing is unpredictable. On the other hand, there is no task-related difference between the respective microstate durations for non-musicians.

The findings with respect to suppression of the N1 amplitude in response to predictable and unpredictable self-initiated (speech) sounds mainly corresponds to the existing literature addressing this topic (e.g., Schafer and Marcus, 1973; Numminen and Curio, 1999; Numminen et al., 1999; Curio et al., 2000; Ford et al., 2001, 2007; Houde et al., 2002; Heinks-Maldonado et al., 2005, 2006, 2007; Martikainen et al., 2005; Bäss et al., 2008; Aliu et al., 2009; Ventura et al., 2009; Baess et al., 2011). Moreover, our results support the idea of an internal forward model deriving predictions about the sensory consequences of a certain motor-action (Wolpert and Kawato, 1998; Wolpert et al., 1998; Wolpert and Ghahramani, 2000; Wolpert and Flanagan, 2001) while tolerating uncertainties regarding the exact physical parameters of the expected sound (Bäss et al., 2008).

However, when comparing MIS of the N1 AEP component in pianists to the N1 suppression observed in musically untrained individuals, we could not confirm our hypothesis regarding the musicians internal forward model generating smaller prediction errors and thus enhanced MIS. As Aliu et al. (2009) described, MIS in the auditory cortex is most likely a learned phenomenon, that is, it is not immediately present but develops over time with repeated and consistent motor-action to sensory-consequence associations. Martikainen et al. (2005) clearly showed that MIS develops after only 60 successive motor-to-sensory couplings. Considering that all participants performed a training block consisting of 60 trials prior to the EEG-experiment, it could be the case that the forward model of musicians learns associations faster causing MIS to develop after fewer trials though the overall magnitude of N1 amplitude suppression obtained during the actual experimental blocks appears to be unaffected.

In fact, our findings concerning the TPA clearly demonstrate that the forward model of musicians actually alters the processing of a self-initiated motor-action's auditory consequences differently compared to the forward model of musical laymen, at least with respect to speech-sounds. The durations of the auditory N1-like template map are shorter in musicians during the first ~200 ms when application of CV-syllables is self-initiated and their outcome in terms of voicing is predictable. This indicates that individuals exposed to intense musical training rely more on their internal forward model's predictions about basic acoustic features of auditory consequences of their own actions. Thus, the needed processing time of the actual auditory input during early stages is reduced, since no further processing is required after a positive match between the sensory goal and actual outcome. This would also explain why this facilitation effect diminishes when speech-sounds are self-initiated but their outcome in terms of voicing is unpredictable. In this case, the forward model's respective predictions are not as reliable causing the need for re-analysis of the actual auditory input which in turn leads to processing times comparable to the ones needed in conditions where speech-sounds are externally initiated. On the other hand, the forward model of musical laymen seems not to exhibit such a facilitation effect with respect to early auditory analysis of speech sounds, indicating that re-analysis is taking place even in case of a positive match.

Our second hypothesis, which was based on the results of our recent study about the processing of voiced and unvoiced acoustic stimuli in musicians (Ott et al., 2011), had also to be rejected. According to the findings of said study, musicians process voiced and unvoiced speech sounds similarly. Hence, we predicted that their forward model generates smaller prediction errors with voiced and unvoiced CV-syllables in the unpredictable task leading to a less prominent attenuation of MIS of the N1 AEP component compared to musical laymen. As our results show, this seems not to be the case. Moreover, we also failed to replicate the corresponding results concerning different physiological activation pattern between musicians and non-musicians in terms of AEP N1 amplitudes and auditory N1-like map durations. In particular, we found in our study of 2011 that N1 amplitudes and N1-like map durations in response to voiced and unvoiced auditory stimuli were comparable in musicians. In contrast, AEP N1 amplitudes were stronger and map durations were longer in non-musicians when processing voiced auditory stimuli relative to unvoiced ones. In the actual study however, both experimental groups showed the same activation pattern with respect to AEP N1 amplitudes and auditory N1-like map durations as the non-musician group in our 2011 study.

Given these discrepancies, the question arises to what extent the results of the two studies are actually comparable. Thus, it is instructive to point out the various differences between the two studies first and foremost with respect to experimental tasks and participants.

First, in our study of 2011 we applied a phonetic categorization task that required active processing of the acoustic properties discriminating voiced and unvoiced phonemes (e.g., VOT, formant-transitions, specific composition of formants, rises/falls in intensity, co-articulations). In opposition to this, the tasks used in our current study emphasize a rather passive/implicit processing of auditory speech stimuli. While the participants attention was mainly focused on maintaining the interval between button presses of ~3 s, active/explicit processing of the auditory stimuli was not required to accomplish the task. At the same time, there are a variety of studies showing that auditory processing can be affected by specific task properties such as cognitive load, difficulty, repetition, and active vs. passive conditions (e.g., Lang and Kotchoubey, 2002; SanMiguel et al., 2008; Karns and Knight, 2009; Rao et al., 2010; Remijn and Kojima, 2010; Ben-David et al., 2011; Kam et al., 2011). From this perspective, it might be the case that the specific differences between musicians and non-musicians with respect to processing of voiced and unvoiced auditory stimuli only become apparent during conditions in which active/explicit processing of auditory input is required.

Second, the musician group in our study of 2011 comprised a high variability with respect to the genre and instruments the participating musicians mainly played whereas the musician group in our study at hand mostly consisted of classically educated pianists. As a matter of fact, it has been substantiated by various studies that musician's processing of sounds highly depends on instrument, genre, performance practice, and level of expertise (e.g., Koelsch et al., 1999; Pantev et al., 2001; Münte et al., 2003; Schneider et al., 2005; Vuust et al., 2005; Nikjeh et al., 2008; Vuust et al., 2009, 2012a,b). It is therefore quite conceivable that the processing of voicing in classic pianists is not even-handedly altered as in musicians of different genres playing different instruments and thus is rather comparable to the processing of voicing in non-musicians.

### Piano condition

In contrast to speech stimuli, we found a different pattern concerning self- versus externally initiated piano tones. Here, N1 amplitudes are significantly enhanced in the MAU relative to the A and MA tasks in both experimental groups whereas no significant suppression occurs when piano sounds are self-initiated relative to when they are externally initiated. In particular, low-pitched piano tones elicit enhanced N1 amplitudes when self-triggered but unpredictable compared to externally triggered low-pitched piano tones (i.e., MAU > A). High-pitched piano tones on the other hand substantially enhance N1 amplitudes when self-triggered and unpredictable relative to self-triggered but predictable (i.e., MAU > MA) ones. Furthermore, we found that durations of the auditory N1-like template map are significantly longer during early auditory processing stages (i.e., 0–202 ms) in the pianist group in the MA task while in both groups the respective microstate durations are generally shorter when piano tones are self-triggered and predictable.

Our results at hand regarding self- vs. externally triggered piano tones seem somewhat contradictory to begin with, considering that most studies examining auditory processing of self- versus externally initiated sounds consistently showed motor-induced suppression of early AEP components (e.g., Schafer and Marcus, 1973; Numminen and Curio, 1999; Numminen et al., 1999; Curio et al., 2000; Houde et al., 2002; Heinks-Maldonado et al., 2005, 2006, 2007; Martikainen et al., 2005; Ford et al., 2007; Bäss et al., 2008; Aliu et al., 2009; Ventura et al., 2009; Baess et al., 2011; Horváth et al., 2012). However, also contrasting findings were reported in several recent pitch-shifted ERP studies of self-produced vocalization (e.g., Behroozmand et al., 2009; Liu et al., 2010; Behroozmand and Larson, 2011; Chen et al., 2012). For example, Liu et al. (2010) compared neural responses to self-triggered pitch-shift stimuli (PSS) to those triggered by a computer during vocalization and listening. Their results showed that unpredictable self-initiated PSS elicited enhanced N1/P2 responses relative to unpredictable externally initiated PSS. Moreover, a similar study by Behroozmand and Larson (2011) reported N1 suppression effects to be strongly affected by the magnitude of PSS and being almost completely eliminated as the magnitude of PSS reached a certain level (i.e., 400 cents; 100 cents = 1 semitone in western music). In sum, these studies suggest that enhanced brain activity can be evoked to distinguish unpredictable self-triggered from unexpected externally triggered auditory stimulation. From this point of view, the general enhancement of the AEP N1 amplitude in response to unpredictable self-initiated piano tones at hand in our current study becomes less surprising. Moreover, larger prediction errors between efference copies and sensory re-afferences result in larger brain responses in the sensory cortex according to the forward model (Wolpert and Miall, 1996). Bearing in mind that the difference between the two piano tones that we used with respect to the fundamental frequency (F0) is fairly large (/C3/, F0 = 130,813 Hz and /C5/, F0 = 523,251 Hz), it might be the case that the enhancement of N1 amplitudes in response to unpredictable piano tones that we observed in the current study is simply due to large mismatches between the forward model's predictions and the actual sensory feedback.

Anyhow, this still does not explain why AEP N1 amplitudes in response to predictable self-initiated piano tones do not exhibit significant suppression relative to predictable externally initiated ones in our current study. Though, a plausible explanation can be derived from the results of a recent MEG study: Aliu et al. (2009) reported suppressed N1m amplitudes in response to short (100 ms), binaurally presented simple 0-delay 1 kHz-tones. In particular, MIS development was clearly present in the left, but did not extend to the right hemisphere. According to the asymmetric sampling in time model (“AST-model”; Poeppel, 2003; Hickok and Poeppel, 2007; Poeppel et al., 2008; Luo and Poeppel, 2012), temporal analysis and integration of “long-scale” auditory input signals (i.e., ~150–300 ms) mainly carrying spectral information preferentially drive the right hemisphere. Hence, it is fairly conceivable that MIS for predictable self-initiated piano tones did not develop to an extent where it can induce observable and statistically relevant differences with respect to AEP N1 amplitudes.

Despite the absence of significant suppression effects of AEP N1 amplitudes, the results of the corresponding TPA reveal alterations of auditory processing of self-initiated piano tones in both experimental groups. Auditory N1-like map durations are significantly shorter in pianists as well as in musical laymen when the piano tones are self-initiated and predictable. Again, this indicates a facilitation effect of accurate sensory predictions by the forward model such as less processing time is needed for early auditory analysis. Moreover, pianists show enhanced N1-like map durations relative to non-musicians in the MA task, suggesting a more refined auditory analysis and increased neuronal representation of self-initiated and predictable piano tones in pianists. This finding fits in well with the results of various other studies showing enhanced auditory processing of complex tones in musicians (e.g., Pantev et al., 1998; Shahin et al., 2005, 2007; Kuriki et al., 2006; Baumann et al., 2008).

However, it is also of importance to briefly address one additional issue at this point: the auditory N1 has been shown to vary as a function of attention. Thus, it might be the case that possible differences in MIS between the groups are corrupted by mere attention effects. Though, the question whether attention influences the N1-suppression effect for self-initiated sounds has recently been addressed by Timm et al. (2013). Their results showed the N1 itself to be affected by attention, but there was no interaction between attention and self-initiation effects. The authors therefore conclude that the N1-suppression effect for self-initiated sounds is independent of attention. Hence, we assume that attention does not account for the effects we observed in the present study.

## Conclusion

Our findings indicate that intense musical training facilitates the processing of predictable self-initiated speech sounds in terms of faster early auditory analysis in the first ~200 ms. In particular, pianists show a comparable auditory N1-like template map duration pattern in response to predictable self-initated speech and piano tones, whereas a substantial reduction of processing time in non-musicians is only present in response to predictable self-initiated piano tones. Moreover, pianists and non-musicians did not significantly differ with respect to suppression of AEP N1 amplitudes neither in response to self-initiated speech sounds nor piano tones. Taken together, our results suggest that pianists rely on their forward model's predictions of sensory outcomes to the same extent when processing speech sounds and piano tones. In contrast, non-musicians seem to analyze predictable self-initiated speech sounds in a more elaborate manner even in the case of a positive match between their forward model's predictions and the actual auditory input. In addition, internal forward mechanisms do not mandatorily lead to suppressed cortical feedback to self-initiated complex sounds but can take beneficial effect with respect to processing time, as indicated by the absence of significant suppression of AEP N1 amplitudes and nonetheless shortened auditory N1-like map durations in response to predictable self-initiated piano tones.

### Conflict of interest statement

The authors declare that the research was conducted in the absence of any commercial or financial relationships that could be construed as a potential conflict of interest.
